# Alteration of a brain network with stable and strong functional connections in subjects with schizophrenia

**DOI:** 10.1038/s41537-022-00305-0

**Published:** 2022-11-04

**Authors:** Liu Yuan, Xiaoqian Ma, David Li, Lijun Ouyang, Lejia Fan, Chunwang Li, Ying He, Xiaogang Chen

**Affiliations:** 1 Department of Psychiatry, and National Clinical Research Center for Mental Disorders, The Second Xiangya Hospital of Central South University, Changsha, China; 2 Mental Health Institute of Central South University, China National Technology Institute on Mental Disorders, Hunan Technology Institute of Psychiatry, Hunan Key Laboratory of Psychiatry and Mental Health, Changsha, Hunan China; 3 Department of Radiology, Hunan Children’s Hospital, Changsha, China

**Keywords:** Schizophrenia, Biomarkers

## Abstract

It is widely accepted that there are some common network patterns in the human brain. However, the existence of stable and strong functional connections in the human brain and whether they change in schizophrenia is still a question. By setting 1% connections with the smallest coefficient of variation, we found a widespread brain functional network (frame network) in healthy people(*n* = 380, two datasets from public databases). We then explored the alterations in a medicated group (60 subjects with schizophrenia vs 71 matched controls) and a drug-naive first-episode group (68 subjects with schizophrenia vs 45 matched controls). A linear support vector classifier (SVC) was constructed to distinguish patients and controls using the medicated patients’ frame network. We found most frame connections of healthy people had high strength, which were symmetrical and connected the left and right hemispheres. Conversely, significant differences in frame connections were observed in both patient groups, which were positively correlated with negative symptoms (mainly language dysfunction). Additionally, patients’ frame network were more left-lateralized, concentrating on the left frontal lobe, and was quite accurate at distinguishing medicated patients from controls (classifier accuracy was 78.63%, sensitivity was 86.67%, specificity was 76.06%, and the area under the curve (AUC) was 0.83). Furthermore, the results were repeated in the drug-naive set (accuracy was 84.96%, sensitivity was 85.29%, specificity was 88.89%, and AUC was 0.93). These findings indicate that the abnormal pattern of frame network in subjects with schizophrenia might provide new insights into the dysconnectivity in schizophrenia.

## Introduction

The past decade has seen an explosion in approaches to noninvasive imaging. The blood oxygen level-dependent signal in functional magnetic resonance imaging (fMRI) identifies intrinsic fluctuations in blood oxygenation, which are indirect markers of neuronal activity^1^. These spontaneous fluctuations occur in various regions of the brain. Functional connectivity (FC) is considered to exist when spontaneous activity in two regions is positively or negatively correlated^2^. This is hypothesized to reflect the broader polysynaptic connections and functional connections between brain regions. Increasingly, there is an increasing body of evidence suggesting that the study of brain function cannot be limited to a single region or individual connections. Rather, it should consider the brain as a whole network organization^3^.

In the construction of a functional brain network, a widely accepted approach is to apply thresholds to determine whether there are connected edges between brain regions. The range of absolute thresholds that were applied had correlation coefficients between *r* = 0.1 and *r* = 0.8, and the range of proportional thresholds was 5–40%^4^. As the threshold value changes, the network incorporates different edges. However, it is a pressing question whether some connections are strong enough to be preserved regardless of the threshold selection. In other words, are there common and strong functional connections in the brain that do not vary with individual differences?

Researchers have identified some common large-scale subnetworks in the human brain^5^, such as the default-mode network (DMN)^6–8^ and the salience network (SN)^9,10^. Studies using graph theory have shown that humans and animals have the same network properties^3,11,12^, such as small-world properties^13,14^ and rich-club nodes^15,16^. Rich-club nodes are hypothesized that a few “rich” brain regions constitute this organization, and they are responsible for distributing a large portion of network communications in the brain^17^. One study of microscale rich-club organization found that in cortical networks, 20% of the neurons contribute up to 70% of the incoming and outgoing information flow^18^. Some researchers have suggested that a few rich nodes ensure efficient neuronal processing at the lowest possible cost^19,20^. This suggests that a few important structures might exist in the brain to provide neurobiological organization and optimal energy allocation^21^. Therefore, we hypothesized that there are some widespread and strong functional connections in the human brain. The coefficient of variation (CV) is a standardized measure of the dispersion of frequency distribution. It is defined as the ratio distribution of the standard deviation to the mean^22^. In the present study, the coefficient of variation was first used to evaluate the dispersion of brain connections and the connections with the smallest CV were defined as stable connections, which meant being little changed across subjects. The stable connections constituted, as we called in this study, a frame network.

Moreover, studies have shown that many mental disorders are likely to be associated with the dysfunction of rich clubs, especially schizophrenia^23,24^. Accumulating evidence from neuroimaging studies has revealed topological abnormalities of brain networks and altered functional connectivity in subjects with schizophrenia^23,25,26^. Schizophrenia is increasingly considered to be a disorder of disconnectivity^27,28^. Thus, we further hypothesized that these frame connections would change in subjects with schizophrenia.

**Fig. 1:**
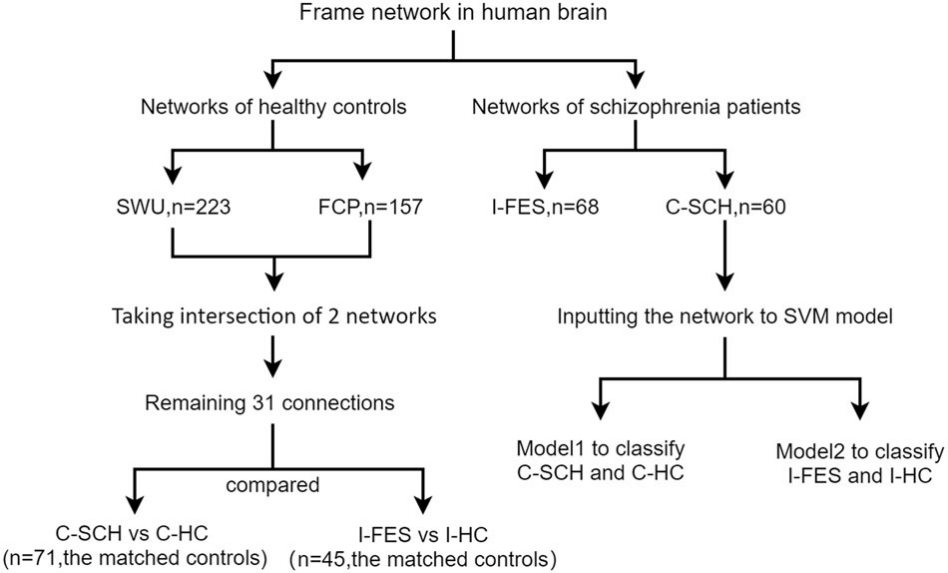
The overall study diagram. Three kinds of subjects were identified in the public databases to construct frame networks. The first group of controls was from the Consortium for Reliability and Reproducibility (CoRR) project at Southwest University (SWU, *n* = 233). The second healthy group was from the 1000 Functional Connectomes Project (FCP, *n* = 157). The medicated subjects with schizophrenia and the matched controls were from the Mind Research Network and the University of New Mexico, funded by the Center for Biomedical Research Excellence (COBRE) projects(C-SCH, *n* = 60; C-HC, *n* = 71). An independent clinical sample was enrolled in this study, including 70 drug-naive first-episode schizophrenia (I-FES) patients and 45 matched controls(I-HC).

To test these hypotheses, we used the fMRI data of 380 healthy subjects from two public datasets to identify frame networks in the human brain. We then compared frame connections of subjects with schizophrenia and their matched controls. Moreover, based on frame networks of schizophrenia, we constructed a support vector machine (SVM) model to classify patients and healthy controls. An independent clinical sample was used to repeat the discriminative power of the patients’ frame network. The study aimed to explore the stable pattern of brain connections in controls and the alterations of these connections in subjects with schizophrenia. Finally, we speculated that this altered pattern could distinguish subjects with schizophrenia and controls from each other. The overall study diagram could see in Fig. 1.

## Results

### Demographic characteristics

The healthy group from the Southwest University project(SWU) included 223 subjects(112 males/111 females) with a mean age of 20.02 ± 1.26 years. Another healthy group was from the 1000 Functional Connectomes Project (FCP), including 157 subjects (53 males/104 females) with a mean age of 21.16 ± 1.81 years. The mean age of medicated subjects with schizophrenia from the Center for Biomedical Research Excellence (COBRE) projects(C-SCH) was 38.77 ± 16.28 years old (including 48 males/12 females) and the matched COBRE healthy controls (C-HC) was about 36.23 ± 11.63 years old(including 48 males/23 females). The independent group of drug-naive patients with first-episode schizophrenia (I-FES) were younger and the mean age was 20.99 ± 5.12 years old, including 44 males and 24 females. The mean age of matched healthy controls (I-HC) was 20.22 ± 3.15 years old, including 25 males and 20 females.

Except for handedness (*p* = *0.01*), there were no significant differences in sex (*p* = 0.12) or age (*p* = 0.27) between C-SCH and C-HC subjects. The C-SCH group had a larger head motion than the C-HC (*p* = 0.002). There were no significant differences in sex (*p* = 0.43), age (*p* = 0.38), education (*p* = 0.26), or head motion (*p* = 0.51) between the I-FES and I-HC groups (see Table 1). However, the age of the I-FES group was significantly lower than that of the C-SCH group (*p* < 0.0001).

**Table 1:** Demographic information.

	Sex (Male/female)	Age (year)	Handedness (R/L/both)	Head motion	Education (year)
SWU (*n* = 223)	112/111	20.02 ± 1.26	-	0.07 ± 0.02	-
FCP (*n* = 157)	53/104	21.16 ± 1.81	-	0.06 ± 0.02	-
C-HC (*n* = 71)	48/23	36.23 ± 11.63	68/1/2	0.15 ± 0.08	-
C-SCH (*n* = 60)	48/12	38.77 ± 16.28	48/10/2	0.20 ± 0.11	-
Degree of freedom: C-SCH vs. C-HC	1	130	1	130	-
*X*^2^ or *F* value	2.55	1.26	9.95	10.54	-
*P* value: C-SCH vs. C-HC	0.12	0.27	0.01^a^	0.002^a^	-
I-FES (*n* = 68)	44/24	20.99 ± 5.12	68/0	0.06 ± 0.03	12.52 ± 2.64
I-HC (*n* = 45)	25/20	20.22 ± 3.15	45/0	0.06 ± 0.03	13.11 ± 2.75
Degree of freedom: I-FES vs. I-HC	1	111	-	111	111
*X*^2^ or *F* value	0.954	0.78	-	0.43	1.29
*P* value: I-FES vs. I-HC	0.43	0.38	-	0.51	0.26

^a^The difference is significant with *p* < 0.05.

### Frame network of each group

**Fig. 2:**
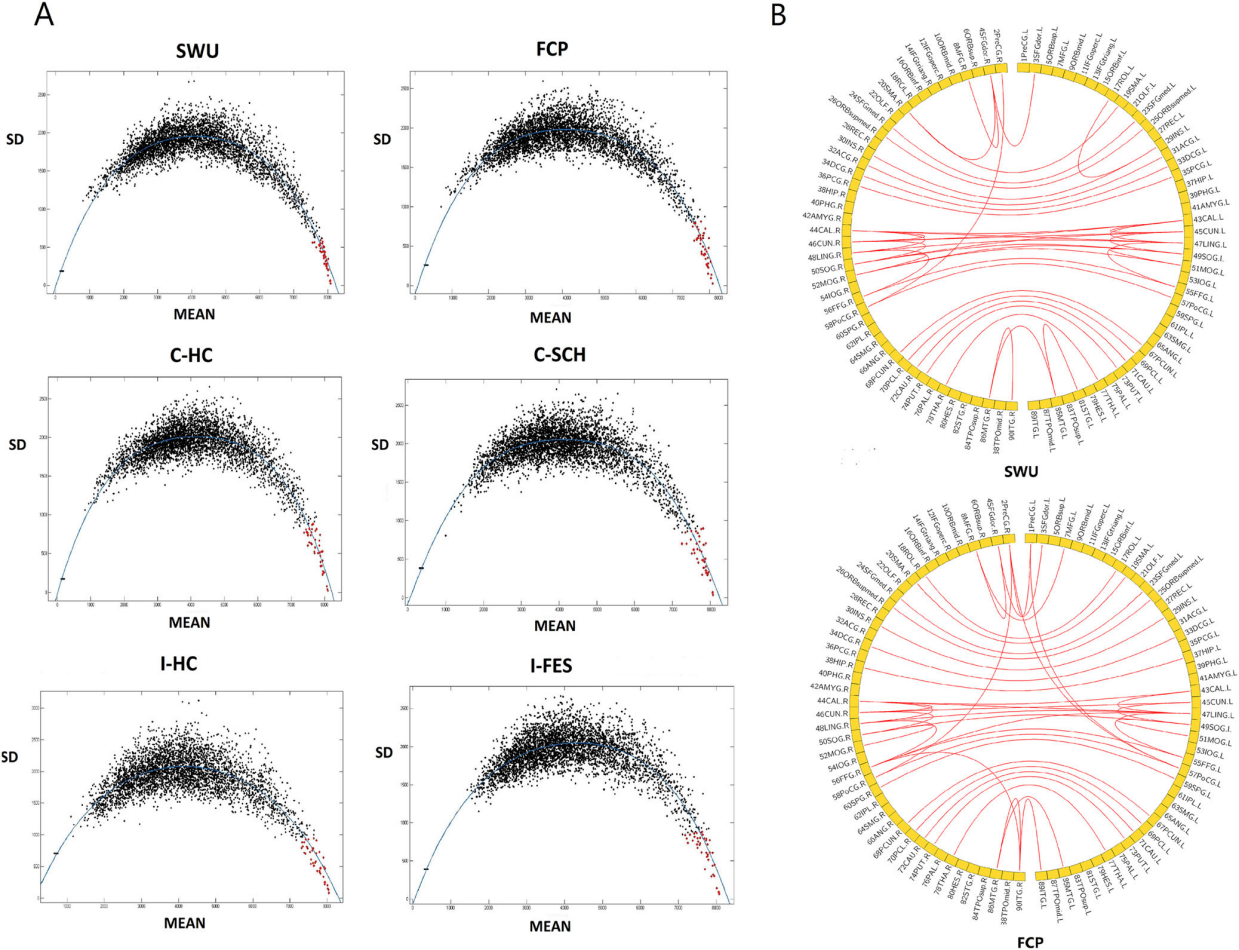
Scatter plots of six groups and the involved brain regions in frame networks of healthy people. The graphs in **A** show a negative correlation between the average ranking and the standard deviation of the six groups. Red dots represent the extracted frame connections. The graphs in **B** show the frame network structure and brain regions involved in the SWU and FCP groups, respectively. The frame connections of the SWU and FCP groups mainly connected the left and right cerebral hemispheres.

Frame networks were similar across the healthy population, with 31 of the 41 frame connections overlapping in the FCP and SWU groups. In those connections with high strength, a negative relation existed between standard deviation(SD) and average ranking (see Fig. 2). This suggested that the stronger a connection, the more stable it was. The frame connections(red dots in scatter plot) we obtained had high connectivity strengths, and they were stable across subjects. Frame networks under the HOA112 template and Craddock200 template yielded similar results. For details, see the supplementary material.

**Fig. 3:**
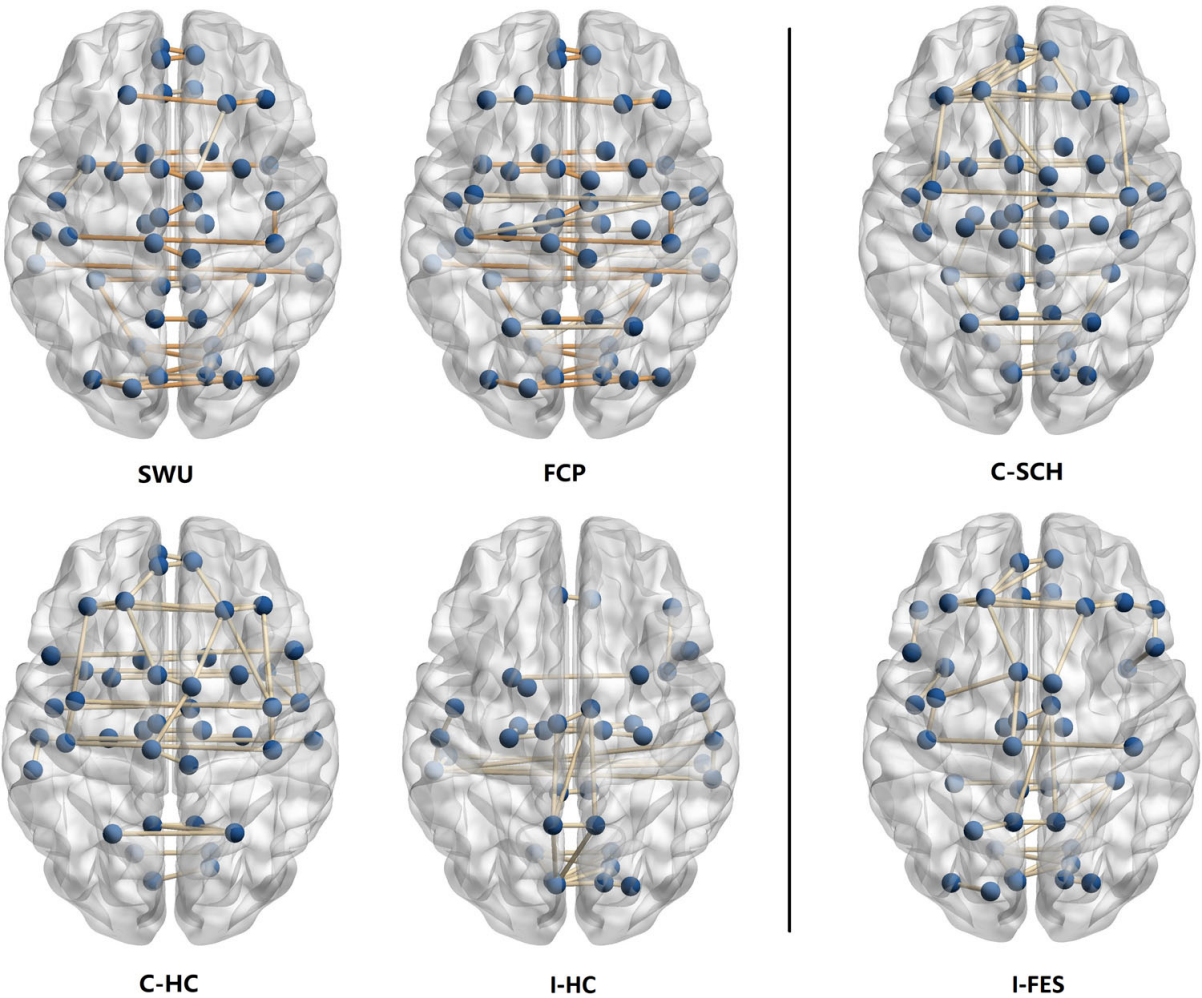
Frame networks of six groups. The orange edges represent the 31 overlapped frame connections in the SWU and FCP groups. Frame networks of the SWU and FCP groups were similar in structure, mainly connecting the left and right cerebral hemispheres. In the patient groups, the frame connections were left-lateralized and focused on the left frontal lobe.

By observing frame networks in the SWU, FCP, C-HC, and I-HC groups, we found that these networks were symmetrically distributed, connecting the left and right hemicerebrums. In contrast, frame networks in the C-SCH and I-FES groups had a lateralized feature, with connections favoring the left frontal lobe (see Fig. 3). Left-sided connections in the C-SCH network were concentrated in the dorsolateral superior frontal gyrus (SFGdor.L) and the frontal middle gyrus (MFG.L). These two nodes had the highest degrees, 6 and 5, respectively. In the I-FES framework network, the connections were concentrated in the SFGdor.L with a degree of 5. ALL brain regions involved in frame networks of healthy people and the C-SCH group can be seen in Table 2. Frame networks in this paper were based on the top 1% connections of the smallest CV. Networks using other thresholds could be seen in the supplementary material.

**Table 2:** Forty-one connections in the frame network of three groups.

SWU	FCP	C-SCH
Frontal_Sup_L-Frontal_Sup_R^a^	Precentral_L-Precentral_R	Precentral_L-Precentral_R
Frontal_Sup_R-Frontal_Mid_R^a^	Frontal_Sup_L-Frontal_Sup_R^a^	Frontal_Sup_L-Frontal_Sup_R
Frontal_Sup_R-Supp_Motor_Area_R	Frontal_Sup_R-Frontal_Mid_R^a^	Precentral_L-Frontal_Mid_L
Supp_Motor_Area_L-Supp_Motor_Area_R^a^	Frontal_Mid_L-Frontal_Mid_R	Frontal_Sup_L-Frontal_Mid_L
Frontal_Sup_Medial_L-Frontal_Sup_Medial_R^a^	Rolandic_Oper_L-Rolandic_Oper_R	Precentral_R-Frontal_Mid_R
Frontal_Mid_Orb_L-Frontal_Mid_Orb_R^a^	Supp_Motor_Area_L-Supp_Motor_Area_R^a^	Frontal_Sup_R-Frontal_Mid_R
Rolandic_Oper_L-Insula_L	Frontal_Sup_Medial_L-Frontal_Sup_Medial_R^a^	Frontal_Mid_L-Frontal_Mid_R
Insula_L-Insula_R^a^	Frontal_Mid_Orb_L-Frontal_Mid_Orb_R^a^	Frontal_Inf_Orb_L-Frontal_Inf_Orb_R
Cingulum_Ant_L-Cingulum_Ant_R	Insula_L-Insula_R^a^	Frontal_Sup_L-Supp_Motor_Area_L
Cingulum_Mid_L-Cingulum_Mid_R^a^	Cingulum_Mid_L-Cingulum_Mid_R^a^	Frontal_Sup_L-Supp_Motor_Area_R
Cingulum_Post_L-Cingulum_Post_R	Hippocampus_L-Hippocampus_R	Supp_Motor_Area_L-Supp_Motor_Area_R
Calcarine_L-Calcarine_R^a^	Calcarine_L-Calcarine_R	Frontal_Sup_L-Frontal_Sup_Medial_L
Calcarine_L-Cuneus_L	Calcarine_R-Cuneus_R^a^	Frontal_Mid_L-Frontal_Sup_Medial_L
Calcarine_L-Cuneus_R	Cuneus_L-Cuneus_R^a^	Frontal_Sup_L-Frontal_Sup_Medial_R
Calcarine_R-Cuneus_R^a^	Calcarine_L-Lingual_L^a^	Frontal_Sup_R-Frontal_Sup_Medial_R
Cuneus_L-Cuneus_R^a^	Calcarine_R-Lingual_L^1^	Frontal_Mid_L-Frontal_Sup_Medial_R
Calcarine_L-Lingual_L^a^	Calcarine_R-Lingual_R^a^	Frontal_Sup_Medial_L-Frontal_Sup_Medial_R
Calcarine_R-Lingual_L^a^	Lingual_L-Lingual_R^a^	Frontal_Mid_Orb_L-Frontal_Mid_Orb_R
Calcarine_R-Lingual_R^a^	Cuneus_L-Occipital_Sup_L^a^	Rolandic_Oper_L-Insula_L
Lingual_L-Lingual_R^a^	Cuneus_R-Occipital_Sup_R^a^	Rolandic_Oper_R-Insula_R
Cuneus_L-Occipital_Sup_L^a^	Occipital_Sup_L-Occipital_Sup_R^a^	Insula_L-Insula_R
Cuneus_L-Occipital_Sup_R	Occipital_Sup_L-Occipital_Mid_L^a^	Cingulum_Ant_L-Cingulum_Ant_R
Cuneus_R-Occipital_Sup_R^a^	Occipital_Sup_R-Occipital_Mid_R^a^	Cingulum_Mid_L-Cingulum_Mid_R
Occipital_Sup_L-Occipital_Sup_R^a^	Lingual_L-Fusiform_L^a^	Cingulum_Post_L-Cingulum_Post_R
Occipital_Sup_L-Occipital_Mid_L^a^	Lingual_L-Fusiform_R	Hippocampus_L-Hippocampus_R
Occipital_Sup_L-Occipital_Mid_R	Lingual_R-Fusiform_R^a^	Calcarine_L-Calcarine_R
Occipital_Sup_R-Occipital_Mid_R^a^	Fusiform_L-Fusiform_R^a^	Cuneus_L-Cuneus_R
Occipital_Mid_L-Occipital_Mid_R	Precentral_L-Postcentral_L	Lingual_L-Lingual_R
Lingual_L-Fusiform_L^a^	Precentral_R-Postcentral_L	Cuneus_R-Occipital_Sup_R
Lingual_R-Fusiform_R^a^	Precentral_R-Postcentral_R^a^	ParaHippocampal_L-Fusiform_L
Fusiform_L-Fusiform_R^a^	Postcentral_L-Postcentral_R^a^	Lingual_L-Fusiform_L
Precentral_R-Postcentral_R^a^	Parietal_Sup_L-Parietal_Sup_R	Lingual_R-Fusiform_R
Postcentral_L-Postcentral_R^a^	Precuneus_L-Precuneus_R^a^	Fusiform_L-Fusiform_R
Precuneus_L-Precuneus_R^a^	Paracentral_Lobule_L-Paracentral_Lobule_R^a^	Precentral_L-Postcentral_L
Paracentral_Lobule_L-Paracentral_Lobule_R^a^	Caudate_L-Caudate_R^a^	Precentral_R-Postcentral_R
Caudate_L-Caudate_R^a^	Putamen_L-Putamen_R^a^	Parietal_Sup_L-Parietal_Sup_R
Putamen_L-Putamen_R^a^	Thalamus_L-Thalamus_R^a^	Precuneus_L-Precuneus_R
Thalamus_L-Thalamus_R^a^	Temporal_Mid_L-Temporal_Mid_R^a^	Paracentral_Lobule_L-Paracentral_Lobule_R
Temporal_Sup_L-Temporal_Mid_L	Fusiform_R-Temporal_Inf_R	Caudate_L-Caudate_R
Temporal_Mid_L-Temporal_Mid_R^a^	Temporal_Mid_R-Temporal_Inf_R^a^	Putamen_L-Putamen_R
Temporal_Mid_R-Temporal_Inf_R^a^	Temporal_Inf_L-Temporal_Inf_R	Thalamus_L-Thalamus_R

^a^The 31 overlapped frame connections in the SWU and FCP groups.

### Alterations of frame connection in patients

An analysis of the 31 frame connections showed that 8 connections in C-SCH were significantly decreased compared to the C-HC subjects(with *p* < 0.05). Nine connections were significantly increased in the I-FES group compared with the I-HC subjects(with *p* < 0.05). C-SCH showed the opposite change to I-FES. The following four connections were significant in both groups: SFGdor.R-MFG.R, left—right supplementary motor areas(SMA), right anterior central gyrus(PreCG.R)—right posterior central gyrus(PoCG.R), left—right PoCG.(see Table 3). In the I-FES group, connectivity between left and right SMA was positively associated with negative symptoms, including the sum score of Panss Negative subscale(r = 0.36, *p* = 0.0487), the score of Emotional withdrawal(r = 0.31, *p* = 0.0487), a score of Poor rapport (r = 0.31, *p* = 0.0487), a score of Lack of spontaneity and flow of conversation(r = 0.33, *p* = 0.0487).

**Table 3:** The strengths of frame connections with a significant difference in C-SCH and I-FES.

	C-SCH	C-HC	C-SCH vs C-HC^b^		I-FES	I-HC	I-FES vs I-HC^c^	
			*P*	T			*P*	T
Frontal_Sup_R-Frontal_Mid_R^a^	1.42 ± 0.37	1.55 ± 0.32	<0.001	−3.76	1.33 ± 0.36	0.78 ± 0.39	<0.001	7.64
Supp_Motor_Area_L-Supp_Motor_Area_R^a^	1.82 ± 0.37	1.91 ± 0.39	0.012	−2.55	1.71 ± 0.42	1.70 ± 0.50	<0.001	5.31
Insula_L-Insula_R	1.43 ± 0.43	1.61 ± 0.39	<0.001	−3.61	1.16 ± 0.40	1.07 ± 0.32	-	-
Cuneus_L-Cuneus_R	1.49 ± 0.36	1.66 ± 0.47	0.001	−2.62	1.53 ± 0.45	1.50 ± 0.39	-	-
Precentral_R-Postcentral_R^a^	1.42 ± 0.40	1.55 ± 0.38	0.009	−2.64	1.26 ± 0.37	0.94 ± 0.49	<0.001	3.78
Postcentral_L-Postcentral_R^a^	1.33 ± 0.46	1.53 ± 0.42	0.001	−3.28	1.28 ± 0.35	0.96 ± 0.47	<0.001	4.16
Precuneus_L-Precuneus_R	1.90 ± 0.36	2.14 ± 0.32	<0.001	−4.50	1.67 ± 0.33	1.62 ± 0.40	-	-
Temporal_Mid_L-Temporal_Mid_R	1.21 ± 0.31	1.36 ± 0.40	0.009	−2.66	1.08 ± 0.39	1.22 ± 0.40	-	-
Frontal_Sup_L-Frontal_Sup_R	1.43 ± 0.38	1.50 ± 0.43	-	-	1.36 ± 0.46	0.56 ± 0.43	<0.001	9.45
Frontal_Sup_Medial_L-Frontal_Sup_Medial_R	1.69 ± 0.42	1.75 ± 0.45	-	-	1.50 ± 0.36	0.82 ± 0.40	<0.001	9.24
Lingual_L-Fusiform_L	1.32 ± 0.35	1.31 ± 0.35	-	-	1.18 ± 0.40	0.88 ± 0.34	<0.001	4.03
Lingual_R-Fusiform_R	1.30 ± 0.35	1.34 ± 0.41	-	-	1.27 ± 0.40	1.05 ± 0.33	0.003	3.08
Paracentral_Lobule_L-Paracentral_Lobule_R	1.47 ± 0.39	1.56 ± 0.32	-	-	1.42 ± 0.47	0.99 ± 0.57	<0.001	4.16

^a^The connections were significant in both groups (with *p* < 0.05). Age, sex, handedness, and head motion were used as covariates and corrected with a false discovery rate (FDR,*q* < 0.05).

^b^Degree of freedom was 130.

^c^Degree of freedom was 111.

### SVM classifier results

Classifier using 31 common connections showed low accuracy(only 56.49%). On the contrary, C-SCH frame network could successfully distinguish patients from healthy people. The model of a 1% threshold network showed the best performance in all thresholds(see the supplementary material). Classifiers mentioned below were based on the 1% threshold network.

In the classifier between C-SCH patients and the C-HC subjects, the accuracy was 78.63% (*p* < 0.001), sensitivity was 86.67% (*p* = 0.002), specificity was 76.06% (*p* = *0.029*), and the area under the curve (AUC) was 0.83 (*p* < 0.001). We found that the connections of SFGdor.L–medial superior frontal gyrus (SFGmed.R) (*w* = 11.00), and Insula_L–Insula_R (*w* = *−*9.30) had the highest absolute weight in this model. The average of all features was 2.96 ± 2.57. This showed that these two edges accounted for a substantial part of the total feature weights. In the classifier between I-FES and I-HC subjects, the C-SCH frame network also had excellent discrimination. The accuracy was 84.96% (*p* < 0.001); sensitivity was 85.29% (*p* = 0.002); specificity was 88.89% (*p* < 0.001) and AUC was 0.93 (*p* < 0.001).

## Discussion

To the best of our knowledge, an altered network with stable and strong functional connections in subjects with schizophrenia is rarely reported. Using the coefficient of variation statistic, we found a frame network with high connectivity strength and stability in the brain of healthy people. It was symmetrical and predominantly connected to the left and right cerebral hemispheres. In subjects with schizophrenia, however, the strength of many frame connections was significantly altered, and all of them were interhemispheric or in the right hemisphere. Compared with the healthy controls, frame network connections of subjects with schizophrenia were more left-lateralized, concentrating on the left frontal lobe, especially SFGdor.L. Individuals with schizophrenia could be effectively distinguished from healthy controls by this left-lateralized network regardless of age and the presence of medication.

A fundamental feature of biological systems is symmetrical organization. The left and right hemispheres of human brains develop with a high degree of evenness at both the anatomical and functional levels^29^. Researchers of genetic effects on human brain connectivity have found that the interhemispheric correlations in white matter connections could be largely attributed to underlying genetic factors, with both higher heritability and strong genetic correlations^30^. Recent research examined local genetic influences on cortical thickness and observed that phenotypic local correlation was highly symmetric between left and right hemispheres^31^. It suggests phenotypic local correlation has a significant basis in shared genetic factors and might be related to early developmental origins of the biological processes. From a genetic perspective, it might partly explain why the frame connections of the human brain were mainly interhemispheric and had high connectivity strength. We suggest that these strong interhemispheric connections might compose the fundamental structures of the functional networks in the brain.

Frame networks was also found in subjects with schizophrenia, which meant that strong and common connections still existed. However, many interhemispheric connections were altered in patients, and the symmetry of the network changed. The frame connections of schizophrenia were concentrated in the left frontal lobe. Altered brain network asymmetry has been linked to development processes^32^ and neuropsychiatric diseases, such as autism and schizophrenia^33–35^. The interhemispheric functional dysconnectivity in schizophrenia has been reported in many studies^36–38^. Neuroimaging studies of auditory verbal hallucinations have suggested that the interhemispheric connectivity between posterior auditory regions is decreased in chronic patients^39^. Lower voxel-mirrored homotopic connectivity of the precuneus and precentral gyrus was seen in subjects with schizophrenia, and it could discriminate patients from controls^40^. We suggest that abnormalities in frame connections, especially interhemispheric connections, are in line with the dysconnectivity hypothesis of schizophrenia^41,42^.

The altered frame connections in both patient groups included SFGdor.R-MFG.R, SMA.L-SMA.R, PreCG.R-PoCG.R, PoCG.L-PoCG.R. Alterations of these brain regions have been widely found in schizophrenia^43–46^. The rs1625579 TT (miR-137 locus) schizophrenia risk genotype was reported to be associated with left dorsolateral prefrontal cortex (DLPFC) hyperactivation^47^. Global brain functional connectivity in the left SFG was increased in subjects with schizophrenia and their unaffected siblings^48^. In the activity of the right and left DLPFC, the posterior part of the SMA was abolished or reduced in subjects with schizophrenia^49^, which was also the case in this study. Researchers have shown that these brain areas are involved in speech and language processing^50,51^, and they contain motor plan and control^52^ and attentional switching and inner speech during language encoding^53^. Generally, most of these brain regions are in anatomical models for heard speech, speech production, and reading^54^. Actually, in this study, negative symptoms, especially language dysfunction in schizophrenia, were significantly correlated with the connectivity between left and right SMA. On the other hand, we observed that frame networks in schizophrenia showed left-ward lateralization, and the connections were concentrated in the left frontal lobe. The left and right hemispheres of the brain display functional specialization in particular cognitive processes^29,55^. Converging evidence has implicated the left hemisphere in language and communication^56,57^. As part of the fabric of language, the left prefrontal cortex was thought to be an inference engine that was based on the left hemisphere’s dominance for language^58–60^. Several studies have reported left prefrontal lobe abnormalities in patients with schizophrenia^61–63^. Combined with the altered connections and the more left-lateralized frame networks in subjects with schizophrenia, it implied that the altered frame networks might be associated with the language processing networks in schizophrenia.

Most strikingly, our findings indicate that the frame network of the medicated group could be effectively discriminated between patients and healthy people, regardless of age and the presence of medication. In the classifier of the COBRE subjects with schizophrenia and controls, the accuracy was 78.63% and AUC was 0.83. The classifier of the independent groups also showed good performance with an accuracy of 84.96%. In addition, the model using 31 common frame Connections was terrible. We speculated the alteration of network structure, instead of connectivity strength, was more prominent in schizophrenia and this left-lateralized frame network might be an independent alteration for schizophrenia. Moreover, the C-SCH frame network had better classification performance with the younger independent samples (I-FES and I-HC). This might indicate that the alteration becomes more pronounced as the disease progresses. Accordingly, a recent study based on the variability of resting-state signal found that most brain regions showed increased left-ward lateralization in patients with schizophrenia, and the lateralization metrics were positively correlated to the age of onset and the duration of the illness^35^. It further suggested that the changed frame networks might be an early network alteration in schizophrenia.

### Limitations and future study

This study was based on resting-state fMRI, without using the structure and cognitive information. Therefore, it might restrict the interpretation of the underlying pathophysiology. In future studies, a comparative approach that integrates data from brain structure with behavior research will be needed to provide significant insight into the frame network and its alterations in schizophrenia. Second, the discrimination power of the C-SCH frame network was repeated in an independent group without verifying the obtained model. The abnormal frame network could be used as an optional classification feature in subsequent research. Despite the similarities in frame networks between the two patient groups, the effect of differences in image acquisition remains a potential confounder. More subjects at different illness stage should be included to explore the discrimination ability of the frame network and analyse the effects of age, disease course, and medication. Third, the medicated group was from COBRE public dataset and the detailed clinical and psychosocial information (such as socioeconomic status, education, family history, etc.) was absent. Therefore, the confounding effects of these factors cannot be excluded. Future studies are needed to further investigate these possibilities. Finally, the left lateralization of the patients’ network and its asymmetry will be measured in the follow-up study.

## Conclusion

We used a new approach to discover the existence of a stable functional network with high strength in the human brain, known as a frame network. In subjects with schizophrenia, many frame connections had altered strength, and their frame networks showed left-ward lateralization with the edges concentrated in the left frontal lobe. This changed frame network showed excellent performance in distinguishing patients from controls, regardless of age and the presence of medication. The alterations in frame networks might reflect the deficits in the language process of subjects with schizophrenia and offer new insights into the patterns of dysconnectivity in this disease.

## Methods

### Participants

We used three public databases of functional magnetic resonance imaging (MRI) data and a set of independent samples. All datasets from public databases were anonymous, with no protected health information included.

Two groups of healthy subjects were identified in the public databases to construct frame networks. The first group was from the Consortium for Reliability and Reproducibility (CoRR) project at Southwest University (SWU). It comprised 235 healthy controls. Among them, one case had no demographic information, five subjects were excluded because of poor image quality, and six subjects were excluded because of excessive head motion. The second group of healthy controls was from the 1000 Functional Connectomes Project (FCP, Beijing_Zang, China). It comprised 198 subjects, but 41 subjects were excluded because of poor image quality.

Functional MRI data from the Mind Research Network and the University of New Mexico funded by the Center for Biomedical Research Excellence (COBRE) projects (http://fcon_1000.projects.nitrc.org/indi/retro/cobre.html) was used to construct a frame network of subjects with schizophrenia(C-SCH) and build a classifier between the patients and the matched healthy controls(C-HC). All subjects were screened and excluded if they had a history of mental retardation, neurological disorder, severe head trauma with more than 5 min loss of consciousness, or substance abuse or dependence within the last 12 months. Diagnostic information was collected using the Structured Clinical Interview used for DSM-IV Disorders. One patient was excluded because of incomplete data, and 11 patients and four controls were excluded because of excessive head motion. As a result, the subjects comprised 60 subjects with schizophrenia and 71 controls.

We used an independent clinical sample to repeat the results of the frame network. 70 drug-naive first-episode schizophrenia (I-FES) patients were enrolled in the outpatient department of the second Xiangya Hospital of Central South University. They were diagnosed through a structured clinical interview according to the DSM-V criteria. Cohen’s kappa was used to assess the inter-rater reliability with κ = 0.85. In addition, 50 healthy controls (I-HC) were recruited by local advertisements. All participants were drug-naïve and right-handed. Subjects were excluded if they had a history of neurological or severe physical diseases, substance abuse, or an IQ <70. The latter was determined using the WAIS-IV^64^. The Positive and Negative Syndrome Scale (PANSS)^65^ was used to evaluate the psychiatric symptomatology of the I-FES subjects. The assessments were conducted by clinical psychiatrists with experience and expertise in PANSS assessment. Inter-rater agreement was 0.97. This study was approved by the Ethics Committee of the Second Xiangya Hospital of Central South University. Written informed consent was obtained from each participant. Two I-FES subjects were excluded because of excessive head motion, and five subjects in I-HC were excluded because of poor image quality.

### Image acquisition

In the healthy group of SWU, the rest data were collected with single-shot full k-space echo-planar imaging (EPI) and the sequence parameters were as follows: TR /TE = 2000/30 ms; slice number = 32; flip angle = 90°; FOV = 220 mm × 220 mm; slice thickness = 3 mm; slice in-place resolution = 3.4 mm^2^ × 3.4 mm^2^; the number of measurements = 242. The imaging parameters for structural MRI data were as follows: TR = 1900 ms; TE = 2.52 ms; flip angle = 9°; slice number = 176; FOV = 256 mm × 256 mm; slice thickness = 1.0 mm; slice in-place resolution = 1.0 mm^2^ × 1.0 mm^2^. In the healthy subjects of the FCP group, the rest image data parameters were as follows: TR = 2 s; slices = 33; time points = 225.

In the group of COBRE subjects, a multi-echo MPRAGE (MEMPR) sequence was used with the following parameters: TR/TE/TI = 2530/[1.64, 3.5, 5.36, 7.22, 9.08]/900 ms, flip angle = 7°, FOV = 256 mm × 256 mm, Slab thickness = 176 mm, Matrix = 256 × 256 × 176, Voxel size = 1 mm × 1 mm × 1 mm, Number of echos = 5, Pixel bandwidth = 650 Hz, Total scan time = 6 min. With five echoes, the TR, TI, and time to encode partitions for the MEMPR are similar to that of a conventional MPRAGE, resulting in similar GM/WM/CSF contrast. Rest data were collected with single-shot full k-space echo-planar imaging (EPI) with ramp sampling correction using the intercomissural line (AC-PC) as a reference (TR: 2 s, TE: 29 ms, matrix size: 64 × 64, 32 slices, voxel size: 3 mm^3^ × 3 mm^3^ × 4 mm^3^).

In the independent set, MRI data were acquired using a 3.0 T magnetic resonance imager (Siemens, Skyra, Germany) equipped with a 16-channel array coil at Hunan Children’s Hospital, Changsha, China. Participants were required to remain still and awake with their eyes closed during the scan. Foam pads and earplugs were provided to minimize head motion. Rest data was collected with single-shot full k-space echo-planar imaging (EPI) and the sequence parameters were as follows: TR /TE = 2000/30 ms; slice number = 36; flip angle = 90°; FOV = 256 mm × 256 mm; slice thickness = 3.4 mm; voxel size = 3.4 mm^3^ × 3.4 mm^3^ × 3.4 mm^3^. For each participant, the functional run contained 250 image volumes in 508 s of scanning time. The structural image was acquired using a high-resolution sequence: TR = 2530 ms; TE = 2.33 ms; flip angle = 7°; slice number = 192; FOV = 256 mm × 256 mm; slice thickness = 1 mm; voxel size = 1 mm^3^ × 1 mm^3^ × 1 mm^3^.

### Image preprocessing

Data from resting-state functional magnetic resonance imaging (rs-fMRI) were preprocessed with Data Processing Assistant for rs-fMRI (running in MATLAB R2013b)^66^. The first ten time points of each image were removed. Then, the images were processed by slice timing, realignment, co-registration to T1 images, and segmentation into gray matter, white matter (WM), and cerebrospinal fluid (CSF). Individual data were transformed into standardized Montreal Neurological Institute coordinates (MNI) space by applying the normalization parameters by DARTEL with a resampling voxel size of (3 mm)^3^. Nuisance covariates regression was carried out, including the CSF, WM signals, and head motion profiles by Friston’s 24-parameter model^67^. Furthermore, nuisance covariates regression was applied to the bad time points, which were defined as any volumes with mean FD (Jenkinson) >0.2 mm^68^, as well as two points before and one point after these volumes. The generated images were then smoothed using a 4 mm^3^ × 4 mm^3^ × 4 mm^3^ full-width at half maximum (FWHM) Gaussian kernel, and the linear trends were removed. Finally, MRI data were band-pass filtered (0.01–0.1 Hz). Subjects with more head motion than 2.5 mm and 2.5° of rotation in any direction were excluded. Using the AAL90 atlas, the functional MRI data of each subject were divided into 90 brain regions. Pearson correlation coefficients between brain regions were calculated, and then a 90 × 90 matrix was obtained by Fisher Z transform.

### Frame network construction of healthy controls and subjects with schizophrenia

**Fig. 4:**
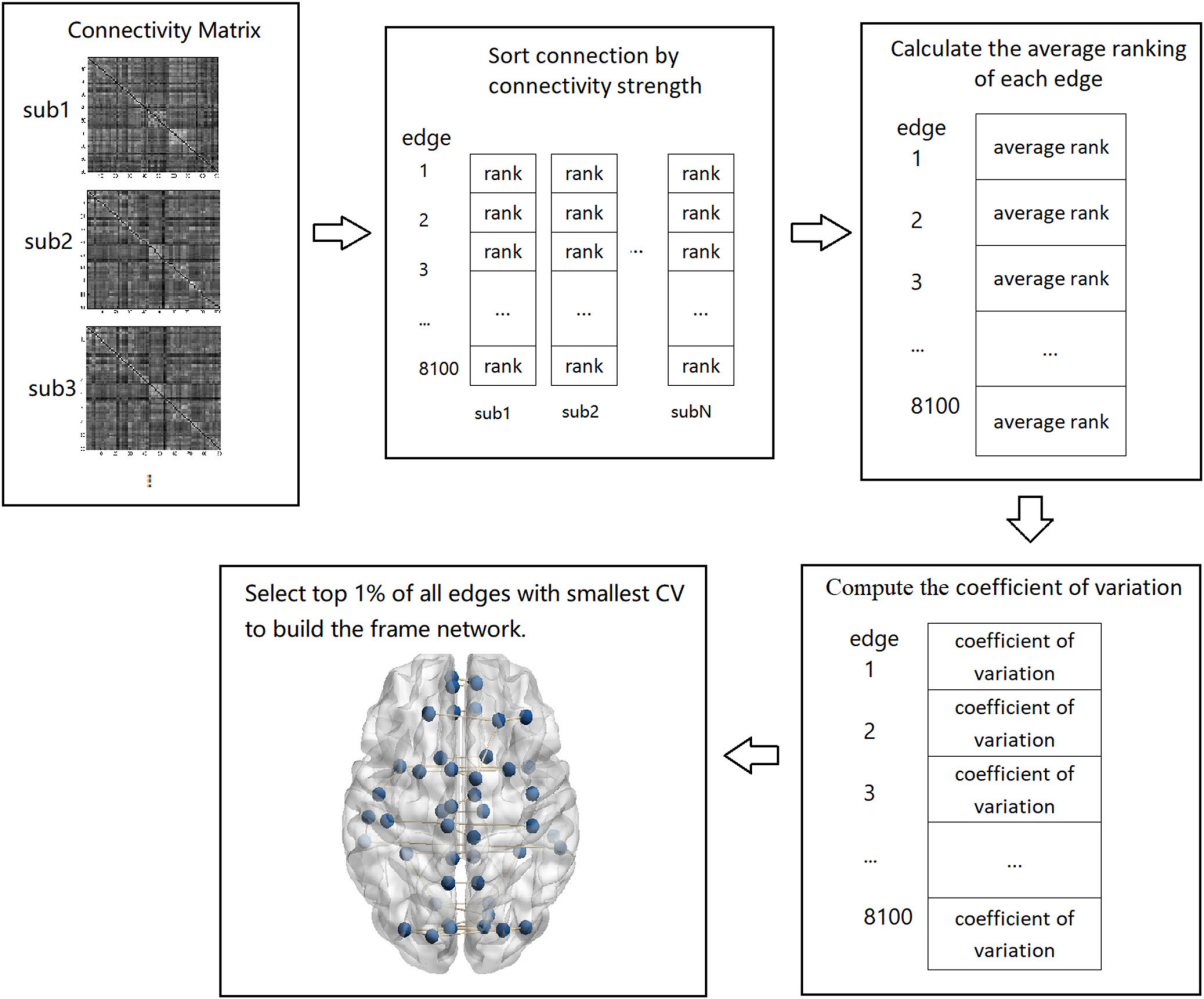
Stages of building frame network. For each subject, the connectivity matrix was converted into a column vector, and the connectivity strength of each edge was sequenced to get the ranking value. The group average ranking of each edge was then calculated. The coefficient of variation of each edge was obtained (standard deviation divided by the mean). The edges with the lowest coefficients of variation (the top 1%, 41 edges) were selected to construct the frame network of the group.

In the 90 × 90 connectivity matrix, the functional connectivity of each connection was obtained. Firstly, for each subject, all functional connections were sorted by connectivity strength and the ranking of each connection was obtained. Secondly, the group average ranking of each edge was calculated. The coefficient of variation was then derived (standard deviation divided by the mean)^69^ to reflect the connection stability. The lower the coefficient, the more stable the ranking of the connection. Top 1% of edges(41 edges, 8100 × 1%/2) with the smallest CV were considered to be stable to constitute the frame network of this group (the flow chat could see in Fig. 4). Frame networks using the HOA112 template^70^ and the Craddock200 template^71^ also were derived to investigate the influence of different atlases. Moreover, we also constructed frame networks using a range of thresholds(from 0.5 to 5%) in healthy controls and patients. The results of the other two templates and different thresholds networks are presented in the supplementary material. In this study, the AAL90 template and 1% threshold were used in the subsequent analysis.

### Comparison of connection strengths between subjects with schizophrenia and controls

By taking the intersection of frame network connections of the SWU and FCP groups, a common network with 31 connections was found in healthy populations. The strength of connections in this network was compared between the COBRE groups of subjects with schizophrenia (C-SCH) and healthy controls (C-HC), and between the independent groups of I-FES and I-HC. The difference is significant with *p* < 0.05. Age, sex, handedness, and head motion were used as covariates and corrected with a false discovery rate (FDR, *q* < 0.05).

Furthermore, the correlations between symptoms and patients’ significantly altered frame connections were analyzed. As the clinical information was absent in the COBRE dataset, we used PANSS scores of the I-FES group to make a correlation analysis. The connectivity of altered connections were correlated with three sum scores (PANSS positive subscore, PANSS negative subscore, and PANSS total score) and 14 sub-scores (positive and negative items), respectively. The difference is significant with *p* < 0.05. FDR correction was performed (*q* < 0.05).

### Classification with the support vector machine

An SVM was operated using LIBSVM software. We selected a frame network of C-SCH to classify C-SCH patients and C-HC subjects. The strengths of the 41 connections in this network were extracted as input to the linear support vector classifier (SVC). A nested cross-validation procedure^72,73^ was used for hyperparameter optimization and estimation of the classifier’s accuracy. The leave-one-out cross-validation (LOOCV) method was performed in the external loop. For each round of LOOCV, one subject was selected as the test set and the remaining subjects as the training set. The models obtained in the training set were applied to classify the testing targets. After 131 iterations (folds), the performance measures were averaged. The indexes of the model’s performance were accuracy, specificity, sensitivity, and AUC. The statistical significance of these indexes was determined by permutation testing (1000 times), with the threshold set as *p* < 0.05. At the same time, to evaluate the discriminative power of the different features, the weights of each feature in all folds were averaged. By the same procedure, C-SCH frame networks under other thresholds were also used to classify C-SCH patients and C-HC subjects, as well as the 31 common connections mentioned above. The results could be seen in the supplementary material.

We selected two other groups (I-FES and I-HC) to repeat the classification ability of the network. The frame network of C-SCH was applied to I-FES and I-HC subjects. By the identical steps, the same features was used to build another SVM model to classify 68 I-FES subjects and 45 controls. The accuracy of the model was corrected after 1000 permutation tests (*p* < 0.05).

### Statistical analysis

Data analysis was performed with SPSS (IBM SPSS Statistics for Macintosh, Version 23.0). Mean FD and demographic variables were compared using analysis of variance (ANOVA) or the chi-squared test. Functional connectivity was compared by two sample *t*-tests with FDR correction (*q* < 0.05). Age, sex, handedness, and head motion were treated as covariates for all statistical comparisons between the groups. Correlations were analyzed using Pearson’s correlation analysis and corrected through FDR (*q* < 0.05). The statistical threshold was set at *p* < 0.05.

## Supplementary information

the supplementary material.

## Data Availability

The SWU group was from the Consortium for Reliability and Reproducibility (CoRR) project at Southwest University (http://fcon_1000.projects.nitrc.org/indi/CoRR/html/swu_4.html). The FCP group was from the 1000 Functional Connectomes Project (FCP, Beijing_Zang, China) (http://fcon_1000.projects.nitrc.org/fcpClassic/FcpTable.html). MRI data in C-SCH and C-HC groups was shared by the Mind Research Network and the University of New Mexico, funded by the Center for Biomedical Research Excellence (COBRE) projects (http://fcon_1000.projects.nitrc.org/indi/retro/cobre.html). The data in the independent groups that support the findings of this study are available from the corresponding author upon reasonable request.
