# Supplementary material for: Alteration of a brain network with stable and strong functional connections in subjects with schizophrenia
Source: Schizophrenia (Heidelb). 2022 Nov 4;8(1):91. doi: 10.1038/s41537-022-00305-0 (PMC9636375; doi:10.1038/s41537-022-00305-0)
Supplement: Supplementary file 1 — the supplementary material. [file 41537_2022_305_MOESM1_ESM.docx]

**Supplementary Material**

## Supplementary Methods

## Frame network construction using different templates

In addition to the AAL90 template, the HOA112 and Craddock200 templates were used in this study. Using the same method, the frame networks of the SWU and FCP groups were extracted. The results showed that the frame networks of the two groups largely overlapped, regardless of the atlas. This suggests that the frame network is stable in healthy populations.

HOA112 template: The connectivity matrix generated from this template was 112×112. We took the top 1% of connections (63 edges, 12,544*1%/2) with the smallest coefficients of variation(CV) to constitute the frame network. Among the 63 frame connections, the SWU and FCP groups overlapped 46. The scatter plots showed that the average rankings of the edges were negatively correlated with the standard deviation (SD). In the frame networks of both groups, most edges connected the left and right cerebral hemispheres.

Craddock200 template: The connectivity matrix generated by this template was 200×200, containing far more functional connections than the other two templates. For comparison, we took the top 0.5% of connections with the smallest CV and obtained a frame network with 40 connections (40,000*0.5%/2). The SWU and FCP groups overlapped in 25 frame connections. The frame networks of the two groups are shown in Figure 1. The scatter plots of the average rankings and the SD are shown in Figure 2. Red dots represent the extracted frame connections.

Figure1 Frame networks of the SWU and FCP groups under two different atlases.


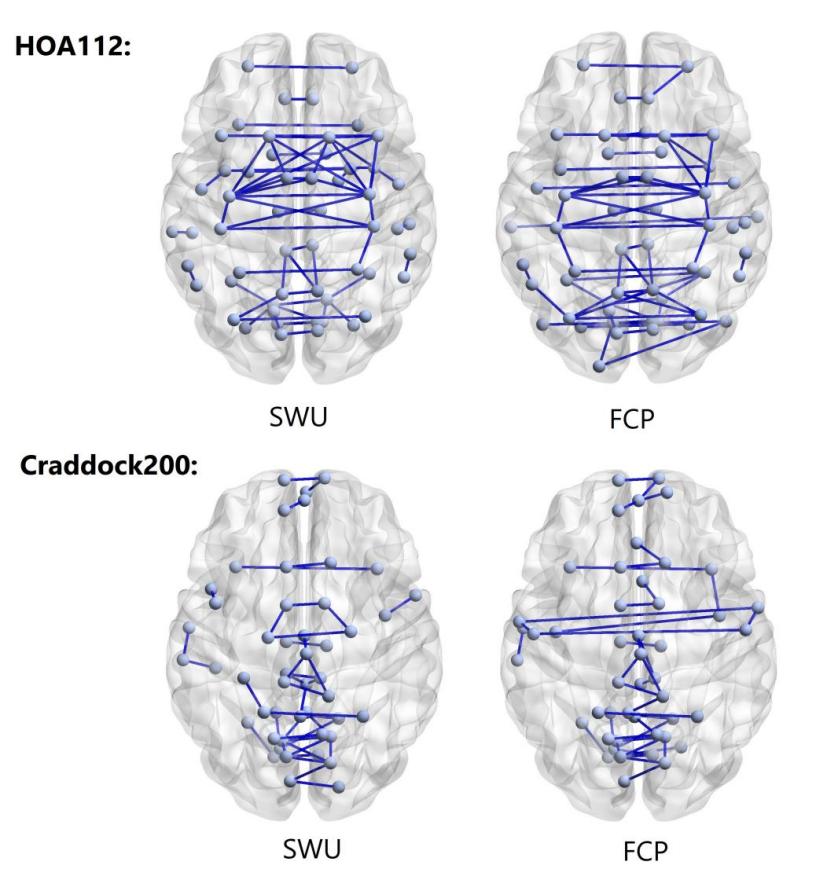


Frame networks of the SWU and FCP groups were similar in structure, mainly connecting the left and right cerebral hemispheres.

Figure 2 Scatter plots of average rankings and the standard deviation under two atlases.


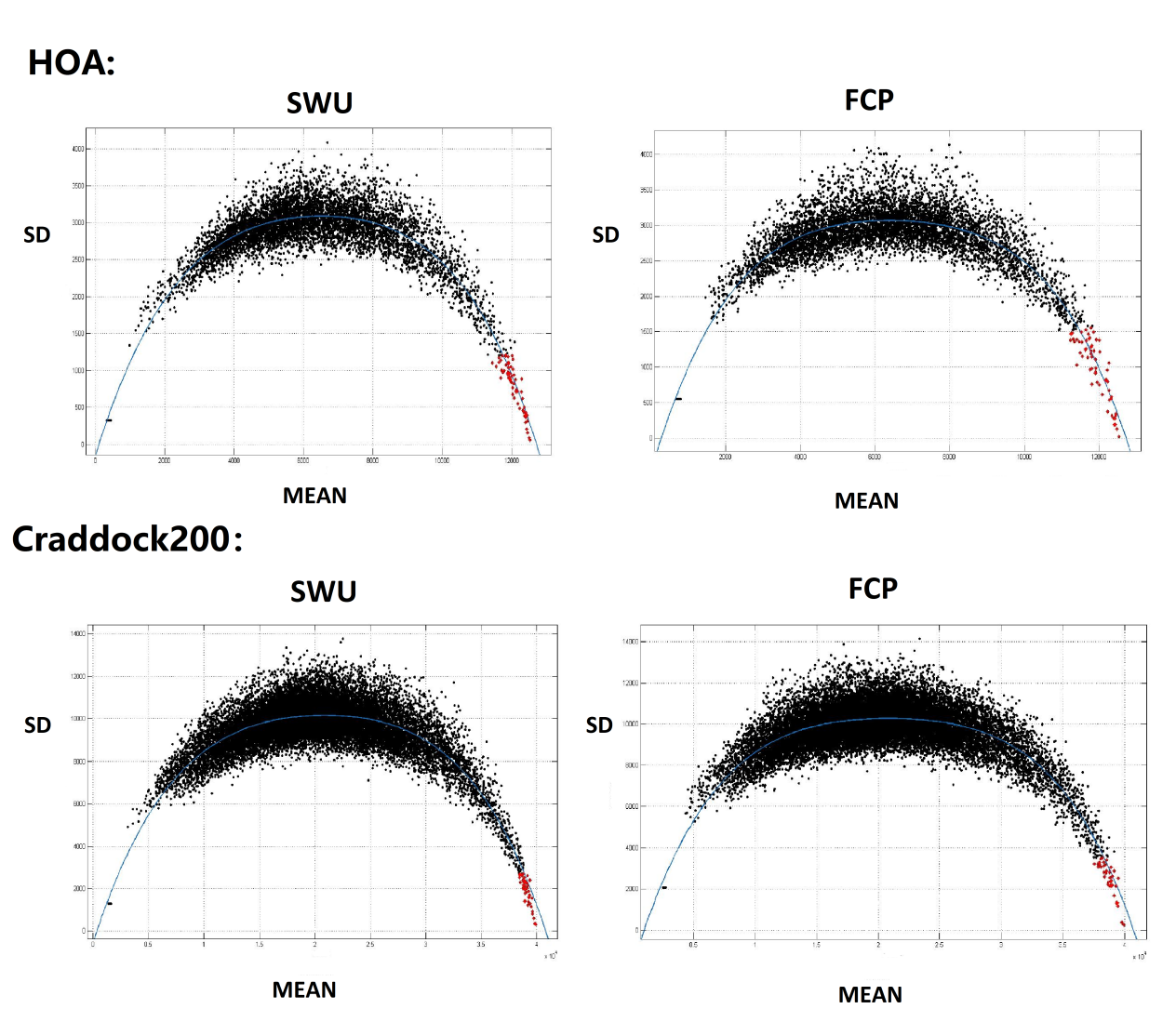


The graphs in A show a negative correlation between the average ranking and the standard deviation of 2 groups using two templates. Red dots represent the extracted frame connections.

## Supplementary Discussion

## Frame network construction using different thresholds

We extracted different frame networks using a range of thresholds in healthy controls and patients(see Figure 3 and 4). It is observed that the network under the 0.5% threshold is sparse, and as the threshold increased, the network become denser. In healthy controls, the networks under different thresholds are symmetrically distributed, connecting the left and right hemicerebrums. In the group of subjects with schizophrenia from the Center for Biomedical Research Excellence COBRE projects(C-SCH), at 0.5-2% threshold, frame networks are concentrated in the left brain, and as the threshold increased, the left lateralization is gradually covered by increased connections. We think tighter threshold is more conducive to explore network alterations in schizophrenia patients. Frame networks under 1% threshold was used in the this study.

Figure3. Different frame networks using a range of thresholds in healthy controls.


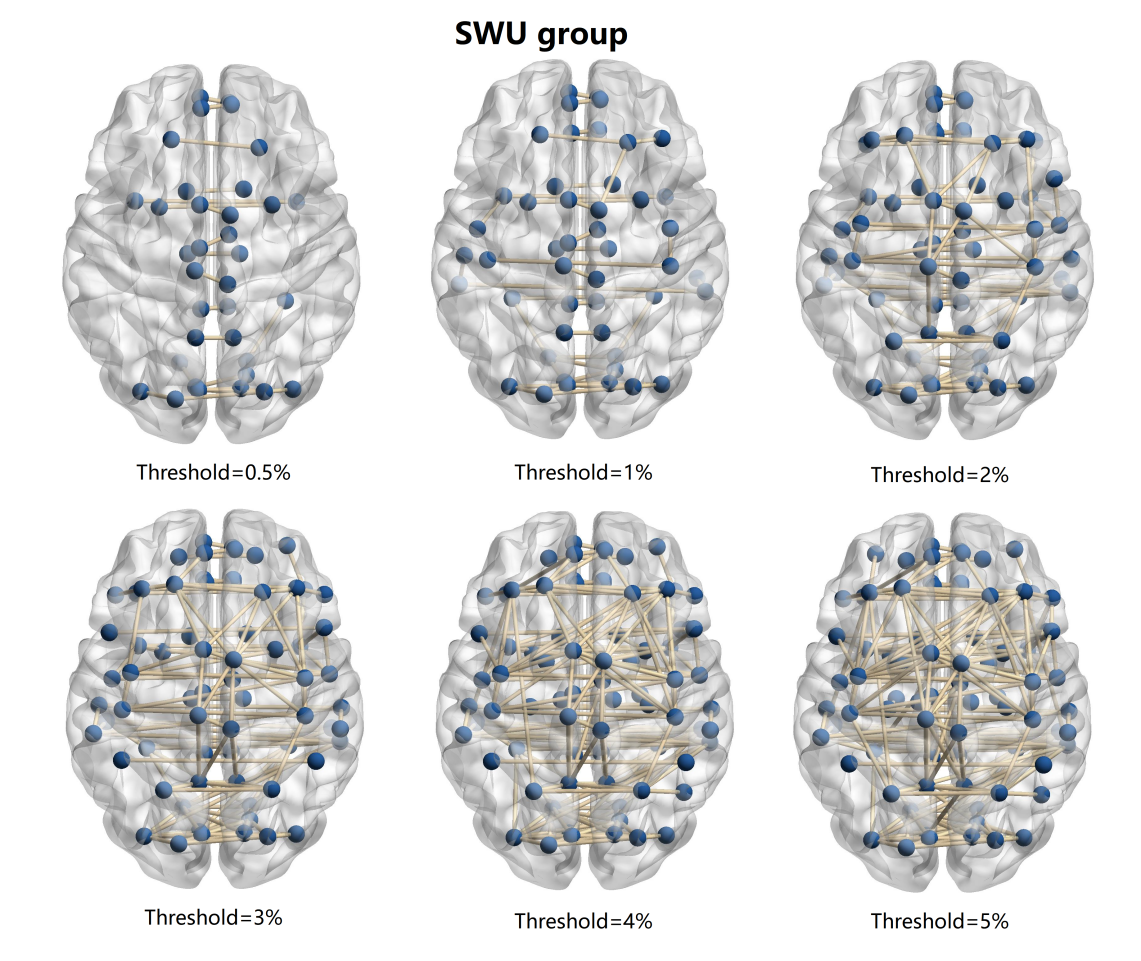


Six frame networks of SWU group under different thresholds. It is observed that the networks under different thresholds are symmetrically distributed, connecting the left and right hemicerebrums.

Figure4. Different frame networks using a range of thresholds in C-SCH group


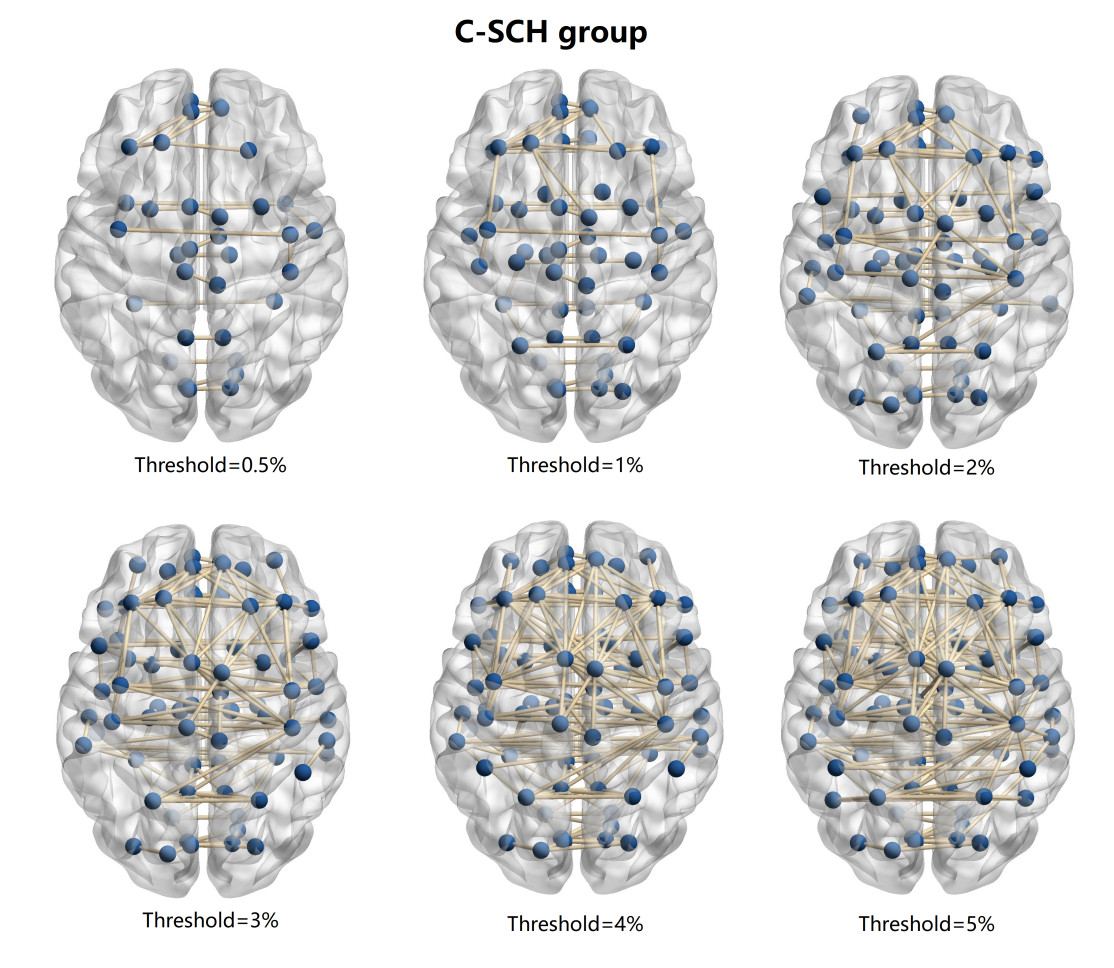


Six frame networks of C-SCH group under different thresholds. At 0.5-2% threshold, frame networks are concentrated in the left brain, and as the threshold increased, the left lateralization is gradually covered by increased connections.

## Classifiers between C-SCH and C-HC groups using different features

In this study, frame network of C-SCH group was used to distinguish patents and controls. Classifiers under different thresholds were constructed and the accuracy indexes were obtained(see Table1 below) . Classifier of 1% frame network showed the best performance. Though patient groups showed altered connectivity strength in 31 common frame connections, the model using these edges was unsuccessful. Frame networks of patients showed left-ward lateralization, which was quite different from controls. We speculated the alteration of network structure, instead of connectivity strength, was more prominent in schizophrenia.

Table1 Performance of different classifiers between C-SCH and C-HC groups

| Classifiers using different features | Accuracy  (%) | Specificity  (%) | Sensitivity(%) | the area under the curve (AUC) |
| --- | --- | --- | --- | --- |
| C-SCH frame network at 1% threshold^1^ | 78.63 | 86.67 | 76.06 | 0.83 |
| C-SCH frame network at 0.5% threshold | 61.07 | 0.65 | 0.63 | 0.66 |
| C-SCH frame network at 2% threshold | 69.47 | 0.70 | 0.73 | 0.78 |
| C-SCH frame network at 3% threshold | 66.41 | 0.79 | 0.62 | 0.75 |
| C-SCH frame network at 4% threshold | 69.47 | 0.70 | 0.68 | 0.75 |
| C-SCH frame network at 5% threshold | 71.76 | 0.73 | 0.73 | 0.82 |
| Using 31 common connections | 56.49 | 0.72 | 0.50 | 0.58 |

^1^：Frame network under 1% threshold was discussed in this paper.
